# Studies of Depression and Anxiety Using Reddit as a Data Source: Scoping Review

**DOI:** 10.2196/29487

**Published:** 2021-11-25

**Authors:** Nick Boettcher

**Affiliations:** 1 Department of Community Health Sciences Cumming School of Medicine University of Calgary Calgary, AB Canada

**Keywords:** depression, anxiety, mental health, Reddit, social media, review

## Abstract

**Background:**

The study of depression and anxiety using publicly available social media data is a research activity that has grown considerably over the past decade. The discussion platform Reddit has become a popular social media data source in this nascent area of study, in part because of the unique ways in which the platform is facilitative of research. To date, no work has been done to synthesize existing studies on depression and anxiety using Reddit.

**Objective:**

The objective of this review is to understand the scope and nature of research using Reddit as a primary data source for studying depression and anxiety.

**Methods:**

A scoping review was conducted using the Arksey and O’Malley framework. MEDLINE, Embase, CINAHL, PsycINFO, PsycARTICLES, Scopus, ScienceDirect, IEEE Xplore, and ACM academic databases were searched. Inclusion criteria were developed using the participants, concept, and context framework outlined by the Joanna Briggs Institute Scoping Review Methodology Group. Eligible studies featured an analytic focus on depression or anxiety and used naturalistic written expressions from Reddit users as a primary data source.

**Results:**

A total of 54 studies were included in the review. Tables and corresponding analyses delineate the key methodological features, including a comparatively larger focus on depression versus anxiety, an even split of original and premade data sets, a widespread analytic focus on classifying the mental health states of Reddit users, and practical implications that often recommend new methods of professionally delivered monitoring and outreach for Reddit users.

**Conclusions:**

Studies of depression and anxiety using Reddit data are currently driven by a prevailing methodology that favors a technical, solution-based orientation. Researchers interested in advancing this research area will benefit from further consideration of conceptual issues surrounding the interpretation of Reddit data with the medical model of mental health. Further efforts are also needed to locate accountability and autonomy within practice implications, suggesting new forms of engagement with Reddit users.

## Introduction

### Background

Interest in studying depression and anxiety using publicly available social media data has grown considerably in the past decade as widespread social media use has dovetailed with the rising global incidence of mental disorders [1,2]. The discussion platform Reddit has become a popular social media data source in this area of study, in part because of the unique ways in which the platform is facilitative of research. This scoping review is about understanding the landscape of research using the social media platform Reddit as a primary data source for studying depression and anxiety. Approach to this research area with a scoping review is supported by 2 points of rationale. First, no work has been done to synthesize the existing research on depression and anxiety using Reddit outside of a small selection of review articles that included Reddit-focused studies under broader topics of social media data and mental health [3-5]. The Reddit platform affords researchers unique methodological opportunities for studying depression and anxiety and warrants a focus that is exclusive of other social media platforms. Second, timely resources are needed to cultivate informed deliberation about the conceptual and ethical dimensions of using publicly accessible social media in research on sensitive health topics, such as mental illness [6], and in public health research and practice more broadly [7]. By illuminating the scope and nature of the research landscape using Reddit data to study depression and anxiety, this review contributes to advancing understandings at the intersection of social media, research practice, and public mental health. The remainder of the *Introduction* section expands on the methodological characteristics of the Reddit platform and grounds the idea of using publicly accessible social media data to study mental health from a historical perspective.

### Methodological Characteristics of the Reddit Platform

Reddit is a social media platform comprising single topic communities called subreddits that are formed, maintained, and participated in by pseudonymous users. Within subreddits, users can submit posts, respond to posts with comments, and reply to comments. Users can also engage with content by granting *upvotes* and *downvotes*, which subsequently inform the default visibility of content to other users. Content moderation is performed by volunteer moderators dually tasked with upholding individual subreddit rules and platform-wide content policies. Reddit harbors a variety of mental health subreddits. Examples relevant to this review, which are established in terms of longevity, size, and user activity, include r/mentalhealth, r/depression, and r/anxiety. Reddit distinguishes itself from Facebook, Twitter, and other health forums with its pseudonymous user system and generous length allowance for posts, comments, and replies. For years, researchers have been attracted to the way these attributes facilitate candid naturalistic expressions and exchanges of mental health information. In turn, Reddit-based studies have a definitive place in the history of studying informal web-based mental health communities [3].

At the outset of this review, it was clear that Reddit facilitates a variety of research approaches and scales of inquiry for studying depression and anxiety. Among the options for collecting naturalistic data, researchers can simply use the search function within the Reddit platform to identify posts, comments, and replies with specified keywords. For researchers interested in larger and more comprehensive data sets, Reddit’s publicly accessible application programming interface (API) can be used to gather batches of data at a time according to the parameters specified in the code. For example, researchers might use the Reddit API to gather all posts, comments, and replies made to a particular subreddit over a defined period and contain a specified keyword. The Reddit API also grants researchers access to select metadata associated with posts, comments, and replies, such as the time of submission and the number of upvotes and downvotes received [8]. A widely used alternative means of accessing Reddit data is called Pushshift, a service created by developer Jason Baumgartner and designed to ingest and archive the entirety of Reddit data on an ongoing basis. As an alternative to the Reddit API, Pushshift offers researchers 2 primary benefits. First, Pushshift allows querying and retrieving historical data that are unavailable via the Reddit API, which enables, for example, the study of a community that was banned from Reddit in the past [9]. Second, Pushshift allows researchers more requests per minute than the Reddit API, shortening the time required to gather large data sets [8,9]. Aside from the Reddit API and Pushshift, researchers interested in studying depression and anxiety using Reddit data may also obtain access to data sets made available by other researchers who have already compiled the relevant data. Having briefly delineated these key methodological entry points for studying depression and anxiety using Reddit data, the remainder of the *Introduction* section provides a general historical contextualization for the study of mental health using naturalistic social media data.

### Studying Mental Health Disorders Using Social Media Data

From a historical perspective, public exchanges of mental health information on social media platforms appeared nearly simultaneously alongside the opportunities for researchers to view and study these exchanges. To illustrate, communications scholar Lomborg [10] has traced the emergence of contemporary social media, both as a *communicative phenomenon* and a *research object*, to 2010. The Lomborg [10] demarcation marks the influx of a broad range of published research examining the intersections of mental disorders and social media, for example, by studying the associations between frequency of social media use and symptoms of depression and anxiety [11]. Within this wider range of inquiry, a research subgenre specifically focused on classifying mental health states using naturalistic data from Twitter and Facebook first appeared around 2013 [4], and comparable studies using Reddit data followed in 2014 [5]. The act of classifying written expressions from social media to make inferences about the mental states of users broke new ground at this time by combining disciplinary orientations and techniques from data science, psychology, and clinical psychiatry. The enthusiasm surrounding novel data sources and methodological possibilities ushered in by contemporary social media also extended to public health more broadly. For example, a 2012 commentary titled *How Social Media Will Change Public Health* predicted public health expanding its scope of practice as it began incorporating new streams of health data, including those related to mental health, which were now *on full display* because of social media [12].

The excitement that marked the early 2010s is in many ways still behind the research efforts to study mental health using social media data. The opportunities for mental illness prevention and mental health promotion remain promising, and the capacities of machine learning (ML) for generating insights from large social media data sets have expanded. However, 2 important sources of temperance arrived later in the decade, with respective origins from social media companies and the academic community. The first is our current *post–public-API age*, referring to the restrictions on public access to social media data via the Facebook and Twitter APIs, which occurred in the wake of the 2016 Cambridge Analytica scandal [13]. Although Reddit has yet to enact comparable restrictions to API access [9], the post–public-API age has renewed emphasis on the foundations of independent social media research by highlighting that access to data is contingent on what private social media companies decide to make or keep available. Second, compelling scholarly arguments for a deeper examination of the conceptual foundations and ethical concerns surrounding the study of sensitive health issues such as mental health using social media data have increased in presence in the latter half of the 2010s [4,10,14]. These key points of historical inflection provide context on how this scoping review of studies on depression and anxiety using Reddit data enters into the brief, fast-moving history of social media’s emergence as an object of dedicated research inquiry.

### Objective and Research Questions

The objective of this scoping review is to determine the scope and nature of research conducted using Reddit as a primary data source for studying depression and anxiety. Specifically, this review proposes to answer the following broad research questions:

1. To what extent have depression and anxiety been studied using the data from Reddit?
2. What are the prevailing analytical practices observed in the included studies?
3. What recommendations for practice have been made by the authors of the included studies?

## Methods

### Overview

Scoping reviews are exploratory review studies conducted to better understand a research area. The unifying activity of scoping reviews can be thought of as *mapping* sources of evidence and key concepts [15]. Similar to the systematic review methodology, scoping reviews require careful planning in advance of collecting the literature. Researchers must articulate a research objective and questions that thread through the search strategy and criteria for assessing sources. However, the purposes for undertaking scoping reviews are typically more flexible than those motivating systematic reviews. Procedures followed for this scoping review were guided by the influential Arksey and O’Malley [16] methodological framework, the Levac [15] elaborations to the Arksey and O’Malley framework, a guidance article from the Joanna Briggs Institute Scoping Review Methodology Group [17], and the PRISMA-ScR (Preferred Reporting Items for Systematic Reviews and Meta-Analyses Extension for Scoping Reviews) checklist [18] for reporting the results of scoping reviews.

### Search Strategy

To identify relevant studies, the search strategy encompassed health science databases MEDLINE, Embase, CINAHL, PsycINFO, and PsycARTICLES and general science databases Scopus and ScienceDirect. Computer science databases ACM Digital Library and IEEE Xplore Digital Library were also searched to capture refereed conference proceedings that may not have been indexed in other science databases [19]. An initial search was performed on October 22, 2020, and the results were imported into Endnote (Clarivate Analytics, Inc) and sorted by databases. To update the review before submitting for publication, a follow-up search was performed on January 22, 2021, with identical search terms and databases. New sources yielded from the follow-up search were identified following the Bramer [20] technique for updating systematic literature searches using Endnote’s deduplication feature. The full Ovid MEDLINE database search strategy is provided in Multimedia Appendix 1.

Studies were also gathered from 2 information sources outside of the academic databases. First, the reference lists of recent review articles, including Reddit-focused studies under the broader topics of social media data and mental health, were hand-searched before the initial database search [3,4,21]. Second, the author set a weekly Google Scholar alert that ran from the day of the initial search to that of the follow-up search and identified newly indexed studies with the word *Reddit* in the title. Studies identified by the weekly alerts were imported into a separate database folder in Endnote during the follow-up search. Google Scholar was restricted to a supplementary search resource because of its limited capabilities for structured searches and issues with transparency and reproducibility of search results [22].

### Eligibility Criteria

#### Overview

Eligible studies used naturalistic data from Reddit as a primary data source and featured an analytic focus on depression or anxiety. Studies were limited to those published in English. To be included at the abstract screening stage, studies had to use the term *Reddit* in their title or abstract and *depression* or *anxiety* in the title or abstract. As familiarity with the subject matter increased, inclusion criteria were further articulated following the *participants concept context* framework suggested by Peters et al [17]. These criteria have been defined in the following subsections.

#### Participants

Participants of the included studies were Reddit users whose publicly available posts, comments, and replies were unobtrusively analyzed as a primary data source for studying depression and anxiety.

#### Concept

Included studies examined depression or anxiety as core concepts, meaning that a conceptual focus on depression or anxiety was specified at the level of methodology. Studies that did not make this methodological specification were excluded even if findings related to depression or anxiety were reported. Aside from the criteria of methodological specification, the concepts of *depression* and *anxiety* were treated inclusively throughout the screening and review steps.

#### Context

The contextual focus of this review is naturalistic data from Reddit. Studies using Reddit to recruit participants for surveys or interviews fall outside this context.

### Study Selection

**Figure 1:**
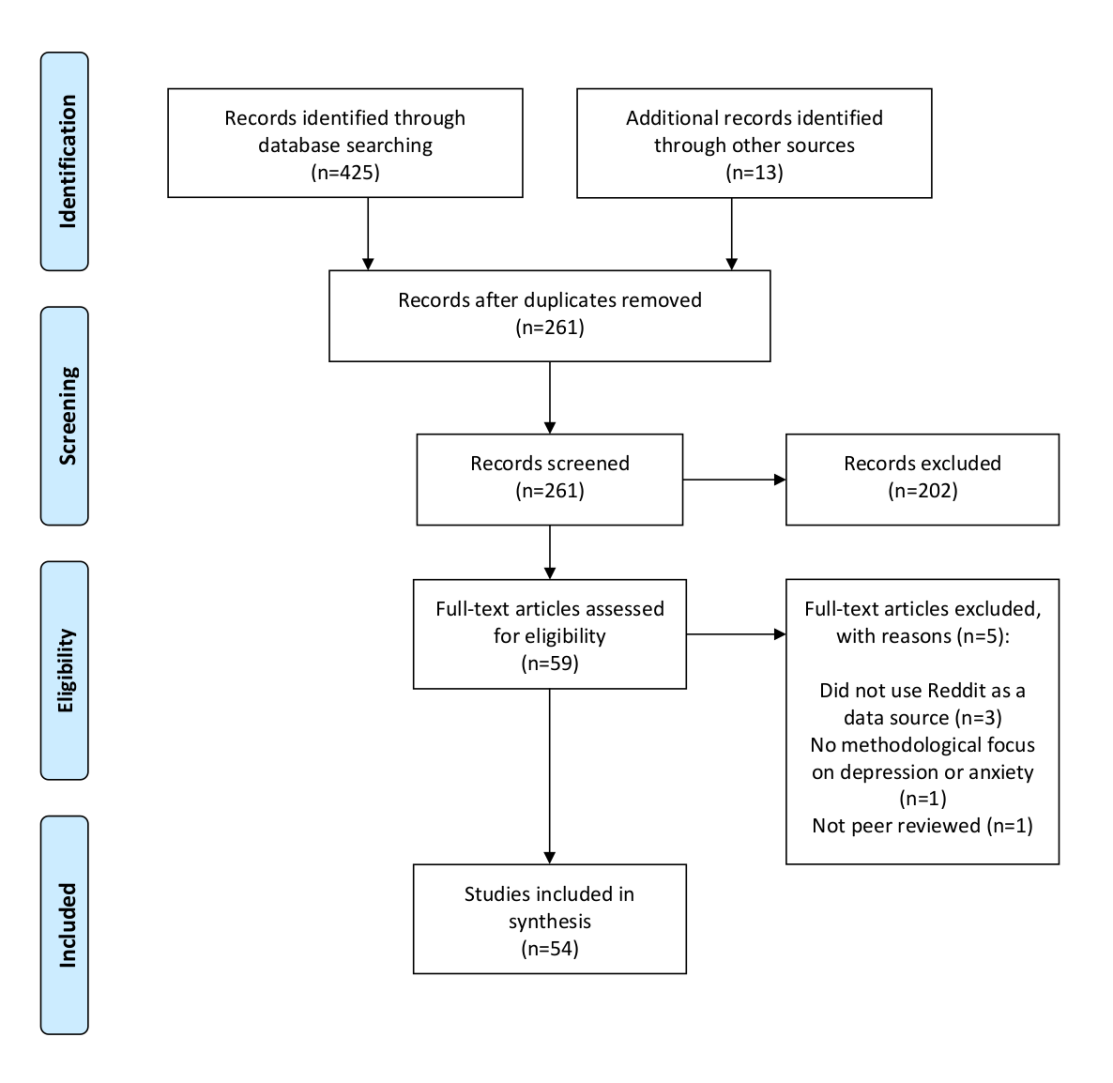
PRISMA (Preferred Reporting Items for Systematic Reviews and Meta-Analyses) flow diagram.

Covidence (Veritas Health Innovation, Ltd) reference management software was used to screen abstracts, review full texts, and chart data from included studies. After duplicates were removed, the author and a second reviewer collaboratively screened the first 10% of abstracts. Then, the author and the second reviewer screened the second 10% of abstracts independently. They then met again to check in and resolve conflicts before independently screening the remaining 80% of the abstracts. This approach resulted in a high degree of agreement between the 2 reviewers (95%), and conflicting screening decisions were resolved by revisiting the abstracts in question and discussing them alongside the study objective and inclusion criteria. A similar approach was taken for the full-text review stage, in which there were no conflicts. A total of 60 additional references were captured in the follow-up search executed on January 22, 2021, and these additional studies were processed through abstract screening and full-text review during collaborative sessions. Data charting for the additional studies was performed using the same form as in the initial studies. The PRISMA (Preferred Reporting Items for Systematic Reviews and Meta-Analyses) flow diagram presented in Figure 1 was backward-corrected to reflect all included sources, including those collected in the follow-up search [20].

### Data Charting

The 2 reviewers (the author and the second reviewer mentioned above) initially met to draft the data charting form. The reviewers used the first iteration of the data charting form to independently chart data from 5 papers before meeting again to revise the form. The reviewers then used the revised form to chart the remainder of the included studies. Information charted from each paper included (1) publication details, (2) study design (objectives and conceptualization of depression or anxiety), (3) methodology (data collection techniques, data set characteristics, data preprocessing, analysis methods, and outcome measures), and (4) results (summary of findings, implications, and recommendations for future research). Early data charting revealed an abundance of studies using ML models, and additional data were charted for these, including the overall type of ML approach, feature extraction methods, and classifier types. For a subset of ML-based studies that were distinctly focused on making predictive classifications of user mental health states, techniques for supporting the ground truth of classification decisions were also charted following a typology of common methods identified by Chancellor and De Choudhury [4]. A formal appraisal of the quality and sources of bias among the included studies was not meaningful to the objective of this scoping review.

### Summarizing and Reporting Results

Once all the data were charted, the included studies were first descriptively summarized according to the date, type of publication (journal article vs conference proceeding), and institutional location of the first author. Then, 4 tables were created to organize the included studies according to the key categories of interest in response to the research objective and questions. Finally, accompanying text was written to support the information contained in the tables through narrative elaboration, illustration, and context. Throughout the reporting process, the imperative of the scoping review methodology to “establish how a particular term is used in what literature by whom, and for what purpose” [23] remained a central concern with respect to depression and anxiety to better understand the broader implications of studying these concepts using data from Reddit.

## Results

### Overview

We screened 425 abstracts for possible inclusion, resulting in 59 (13.9%) full-text articles being assessed for eligibility. Of the 59 papers, 5 (8%) full texts were excluded at the full-text stage, leaving 54 (92%) studies to be included in the review (Figure 1) [24-77]. The included 54 studies were conducted between 2014 and 2020 and comprised of 31% (17/54) journal articles and of 69% (37/54) refereed conference proceedings. The claims of the fast-advancing nature of this research area are not hyperbole, as 17% (9/54) of the included studies were published and newly indexed in the 3-month interval between the initial and follow-up database searches. The first authors of the included studies were associated with institutions from 21 different countries, and the United States was the most represented among these, with 30% (16/54) of the studies.

A group of 16 included studies was unique in its association with the Conference and Labs of the Evaluation Forum (CLEF), an annual independent peer-reviewed information systems conference organized around experimentation on shared tasks. In 2017, CLEF introduced the *eRisk* shared task, which invites entrants to create experimental models for predicting mental health risks using social media data sets supplied by the conference organizers [78]. Owing to the fact that CLEF eRisk tasks across the years 2017-2020 used data sets collected from Reddit and featured a focus on depression detection, our search strategy captured the studies associated with eRisk tasks held in 2017 (8/54, 15%), 2018 (5/54, 9%), 2019 (1/54, 2%), and 2020 (1/54, 2%). This group has been referred to as the *CLEF eRisk studies* throughout the *Results* and *Discussion* sections as further observations have been made about the distinct contributions of the eRisk shared tasks to the overall landscape of research using Reddit data to study depression and anxiety. Following the Figure 1 PRISMA flow diagram, tables and corresponding results have been presented according to 4 key study characteristics: (1) mental health conditions of focus, (2) data collection approaches, (3) analytic focus, and (4) practical implications. The first of these study characteristics has been presented in Table 1, which has organized the included studies according to their depression versus anxiety focus while also conveying an overall picture of the mental health conditions studied.

**Table 1:** Mental health conditions of focus (N=54).

Mental health condition of focus		Number of included studies with focus on condition, n (%)	References
**Depression (n=32)**			
	No additional conditions studied	20 (63)	[24-27,29,30,34-38,45,46,57,58,63,64,69,70,74]
	Eating disorders	5 (16)	[28,31-33,39]
	Bipolar disorder	4 (13)	[41,59,65,77]
	Context: postpartum depression	3 (9)	[56,67,75]
	Schizophrenia and psychosis	1 (3)	[59]
	Self-harm	1 (3)	[76]
**Depression and anxiety (n=19)**			
	Bipolar disorder	14 (74)	[40,42,44,47,49,50,53-55,62,66,71-73]
	Suicidal ideation	8 (42)	[44,47,49,50,55,62,66,73]
	Borderline personality disorder	8 (42)	[49,50,53-55,62,66,73]
	Neurodevelopmental conditions^a^	8 (42)	[49-51,54,55,71-73]
	Schizophrenia and psychosis	7 (37)	[42,49,50,53-55,66]
	Posttraumatic stress disorder	6 (32)	[28,42,51,53,60,66]
	Self-harm	5 (26)	[42,49,50,62,66]
	Eating disorders	4 (21)	[43,53,62,73]
	Depersonalization and derealization disorder	3 (16)	[42,62,66]
	Substance use	4 (21)	[44,55,62,73]
	Dementia	2 (11)	[49,65]
	Context: rheumatoid arthritis	1 (5)	[61]
**Anxiety (n=3)**			
	No additional conditions studied	2 (67)	[52,68]
	Bipolar disorder, borderline personality disorder, neurodevelopmental conditions^a^ schizophrenia, self-harm, and substance use	1 (33)	[48]

^a^The category *neurodevelopmental conditions* refers to studies in which authors specify a focus on autism, attention deficit hyperactivity disorder, and/or Asperger syndrome.

### Mental Health Conditions of Focus

Of the 54 studies, depression was researched in 51 (94%) studies, including 20 (37%) studies that exclusively focused on depression. Meanwhile, anxiety was researched in 43% (23/54) of studies, and only 4% (2/54) of studies examined anxiety exclusively. The relative prominence of depression-focused studies can be partially explained by the collection of 16 CLEF eRisk studies, all of which focused on the detection of depression. Table 1 also captures 31% (5/16) of the CLEF eRisk studies that reported participation in a separate shared task for the early detection of anorexia in addition to the 2018 shared task for depression detection. Overall, the abundance of conditions studied in addition to depression and anxiety signals research interest in comparative studies of mental health conditions using Reddit data. Many of the additional conditions were related to depression and anxiety, as exemplified by the inclusion of bipolar disorder in 35% (19/54) of the studies. However, a particularly wide breadth of conditions of focus was noted in some studies that used Reddit data to investigate mental health phenomena as diverse as autism and dementia within a single study while also focusing on depression and anxiety. In summary, depression was researched to a greater extent than anxiety by a wide margin, and it is clear that the Reddit platform facilitates research methodologies designed for simultaneous examinations of multiple mental health conditions. In order to better understand the methodological execution of included studies, the approaches taken to data collection have been presented in Table 2.

**Table 2:** Data collection approaches (N=54).

Data collection approaches (data set category) and data source			Included studies, n (%)		References
**Studies using original data sets (n=24)**					
	Reddit API^a^	12 (50)		[42,44,46,52,60,62,63,68,71,72,75,77]	
	Pushshift	10 (42)		[47,49,50,53-55,58,59,66,76]	
	Reddit search function	1 (4)		[61]	
	Google search	1 (4)		[56]	
**Studies using a premade data set (n=21)**					
	CLEF^b^ eRisk 2017 data set	8 (38)		[24-26,29,34-37]	
	CLEF eRisk 2018 data set	5 (24)		[27,31-33,39]	
	CLEF eRisk 2019 data set	1 (5)		[38]	
	CLEF eRisk 2020 data set	1 (5)		[30]	
	Multiple CLEF eRisk data sets	1 (5)		[28]	
	Data set from Yates et al [76]	3 (14)		[45,69,74]	
	Data set from Gkotsis et al [50]	1 (5)		[48]	
	Data set from Pirina and Çöltekin [63]	1 (5)		[70]	
**Studies with multiple data collection approaches (n=4)**					
	Reddit API and Pushshift.io	1 (25)		[57]	
	Reddit search function and Reddit API	1 (25)		[67]	
	Reddit API plus data set from Pavalanathan and De Choudhury [62]	1 (25)		[41]	
	CLEF eRisk 2017 data set and data set from Yates et al [76]	1 (25)		[64]	
**Studies with unclear data collection approach (n=5)**					
	Data collection not clearly described	5 (100)		[40,43,51,65,73]	

^a^API: application programming interface.

^b^CLEF: Conference and Labs of the Evaluation Forum.

### Data Collection Approaches

Table 2 organizes the included studies according to how researchers approached data collection. Approximately 44% (24/54) of studies reported creating original data sets, and 22% (12/54) of studies did so by accessing Reddit’s official API. Another 19% (10/54) of studies created original data sets by accessing Pushshift, an archived corpus of Reddit data managed by the developer Jason Baumgartner and sometimes signaled within studies as the Reddit *data repository* [47] or *data dump* [49]. Perhaps unsurprisingly, the 4% (2/54) of studies that collected original data sets using neither the Reddit API nor Pushshift were qualitative studies using smaller data sets. To illustrate, Park et al [61] used the Reddit search function to collect a sample of 81 discussion threads for qualitative descriptive analysis, whereas the Maxwell [56] qualitative study used Google Search to identify a single discussion thread consisting of 1 post and 294 comments, with which Maxwell et al [56] performed an in-depth thematic analysis.

Researchers used premade data sets in 39% (21/54) of the studies. Of the 54 studies, data sets for all 16 (30%) included CLEF eRisk studies were categorized as premade as eRisk participants were supplied with Reddit-based data sets created by competition organizers. A total of 2 observations bear mentioning here about the nature of these CLEF eRisk data sets. First, year-by-year iteration was reflected in the 2018, 2019, and 2020 CLEF eRisk data sets using portions of data sets from the preceding year as testing data for participants to tune their classification systems. Second, 9% (5/54) of studies identified using CLEF eRisk data sets after the official timeline of respective annual tasks. The availability and continued uptake of CLEF eRisk data sets is notable as it allows researchers to take their time to study and attempt to outperform the best-performing entries. Aside from the CLEF eRisk studies, the existing data sets accessed by researchers were made available through previously published studies. The most influential among these was the *Reddit Self-Reported Depression Diagnosis* data set, introduced in an included 2017 study by Yates et al [76] and subsequently cited as a primary data source in 7% (4/54) of other studies.

Of the 54 studies, 5 (9%) studies collected data from Reddit using multiple means. An inventive example of multiple approaches to data collection was noted in the study by Shatte et al [67], which used Reddit data to examine social media markers of postpartum depression in fathers. Shatte et al [67] began data collection using the Reddit search function to identify a cohort of 365 Reddit users who made birth announcements in a subreddit for fathers from 2016 to 2018. Then, these authors accessed the Reddit API to collect all posts and comments made by these 365 users in the 6 months before and after each user’s birth announcement, resulting in a data set of 67,796 posts and comments. Finally, they analyzed changes in depressive language and discussion topics following each user’s birth announcement [67]. Combinatory approaches to data collection, such as those deployed by Shatte et al [67], reflect the unique methodological possibilities afforded by the Reddit platform.

Finally, 9% (5/54) of studies were inexact in describing how the data from Reddit were accessed. For example, the De Alva et al [40] study described *selecting* 32 posts containing keywords from 6 mental health subreddits without stating how the researchers searched for the keywords. Other examples included references to data being *crawled* [73], *downloaded* [65], and *collected* [43] without further specification and an uncited reference to a previously used data set [51]. Having conveyed an overview of data collection approaches, Table 3 presents the analytic focus of the included studies to better understand how researchers used their data sets to study depression and anxiety.

**Table 3:** Analytic focus of included studies (N=54).

Analytic focus (general focus and specific focus)		Included studies, n (%)	References
**Focus on predictive mental health classification (n=36)**			
	Binary classification of user mental health	27 (75)	[24-29,31-37,39,45,51,63,64,67-72,74-76]
	Severity-focused classification of user mental health	2 (6)	[30,38]
	Disclosure-focused classification of user mental health	1 (3)	[42]
	Multilabel classification of user mental health	4 (11)	[50,53,54,65]
	Subreddit-level mental health classification	2 (6)	[41,48]
**Focus on mental health language and interactions (n=18)**			
	Subreddit-level analysis	12 (67)	[43,44,46,49,55,57,60,66,73,77]
	User-level analysis	3 (17)	[52,58,62]
	Discussion-level qualitative analysis	3 (17)	[40,56,61]
	Multilevel analysis	2 (11)	[47,59]

### Analytic Focus

Table 3 presents the primary analytic focus of the included studies organized broadly into studies that used Reddit data to (1) make predictive classifications of user mental health states and (2) analyze mental health language and interactions. Within the 2 general categories, studies were further organized into subcategories according to conceptual focus and scale of inquiry.

#### Studies Focused on User Mental Health Classification

Approximately 67% (36/54) of studies used data from Reddit as the basis for predictive mental health classification using ML. The most popular ML approaches were deep learning (DL; 11/36, 30%), supervised ML (6/36, 17%), and combinations of DL and supervised machine (9/36, 25%).

Researchers took a range of approaches to process naturalistic Reddit data into features for use in their ML systems and, in many cases, combined and compared the feature sets within a single study. The most represented features among the classification studies were based on categories of words from pre-existing mental health lexicons and data sets (14/36, 39%). Other common features included n-grams (9/36, 25%), bag-of-words (9/36, 25%), and term frequency–inverse document frequency vectors (9/36, 25%). ML classification was most frequently performed with a neural network (17/36, 47%), support vector machine (10/36, 28%), random forest (7/36, 19%), and logistic regression (6/36, 17%) classifiers. Of the 36 classification studies, 26 (72%) reported on binary classification, with decisions about the mental health states of Reddit users framed in terms of *yes or no* (12/36, 33%) and *at risk or not at risk* (14/36, 39%). Approximately 11% (4/36) of studies performed multilabel mental health classification with DL models that predicted *which* mental health problem was represented in the Reddit user text. Multilabel classification appears to be a forerunning analytic focus, as all 4 studies in this category were published in 2020.

A focus on the severity of depressive symptoms was observed in 6% (2/36) of the classification studies associated with the 2019 CLEF eRisk shared task on early depression risk detection. The authors of these severity-focused experiments were given a data set of Reddit posts and asked to ordinally classify users’ depression as mild, moderate, or severe by transposing inferred signs of depression into responses to the Beck’s Depression Inventory questionnaire [79]. Another approach to user classification was found in the Balani and De Choudhury [42] study in which user posts from mental health subreddits and control subreddits were classified according to self-disclosure, defined as the degree to which users revealed personal information and vulnerable thoughts, beliefs, and experiences. Of the 36 classification studies, 2 (6%) studies endeavored to perform mental health classification at the subreddit level. These included the Gaur et al [48] study, which mapped the aggregate content of 15 mental health subreddits to diagnostic categories from the Fifth Edition of the Diagnostic and Statistical Manual of Mental Disorders, and the study by Bagroy et al [41], which gathered 43,468 posts from *mental health* and *control* subreddits to train and test a model for classifying broad trends in expressions of mental health and distress in 109 university subreddits.

#### Ground Truth in User Mental Health Classification Studies

Among the 36 mental health classification studies, broad trends were noted in the provision of ground truth, or in other words, the baseline by which predictive classifications of the mental health states of Reddit users could be considered valid [14]. Although the following trends are outlined here individually, they usually appeared in some combination within single studies. Researchers targeted posts from mental health subreddits in 75% (27/36) of studies and collected user posts deemed to express self-disclosure of a mental health diagnosis in 69% (25/36) of studies. The ground truth of predictive claims was supported with control data in 78% (28/36) of studies. For example, the Shen and Rudzicz [68] study used ML to classify anxiety in user posts with a data set of 9971 posts from 4 anxiety subreddits and 12,837 posts from 25 control subreddits deemed unrelated to mental health. Human annotators contributed to labeling data in 75% (27/36) of studies and were variously described as layperson annotators [76], raters familiar with Reddit and its mental health communities [42], Amazon Mechanical Turk workers [65], 2 mental health domain experts [48] a clinical psychologist [67], a social media expert and clinical psychologist duo [41], and simply human annotators [53,66]. Of the 36 mental health classification studies, 14 (39%) studies incorporated external mental health data sets into data labeling procedures to support the ground truth of classification. External data set sources ranged from Wikipedia [36], Twitter [37], and AskAPatient [65] to formalized medical sources, including the Unified Medical Language System [31], the International Classification of Diseases, 10th Revision [48], and the Fifth Edition of the Diagnostic and Statistical Manual of Mental Disorders [48,69].

In the 16 CLEF eRisk studies, ground truth relied on the data sets and parameters of analysis provided by eRisk shared task organizers and featured a combination of the community participation, self-disclosure, and control data trends outlined above. Notably, the 2019 and 2020 shared tasks took an additional step in which CLEF organizers contacted Reddit users whose posts and comments were collected for the shared task data set and asked them to fill out a validated depression questionnaire. User-completed questionnaires were then used as ground truth for assessing the performance of entrants who attempted to fill out the same questionnaires using only a curated history of Reddit posts and comments from each user [80]. Although user-completed depression questionnaires supply a more traditionally valid conception of ground truth than annotated disclosures of depression diagnoses in Reddit posts, the solicitation of these questionnaires marks a disjuncture from the passive analytic focus that otherwise characterized the mental health classification studies.

#### Studies Focused on Mental Health Language and Interactions

In distinction to studies focused on classifying the mental states of users, 33% (18/54) of studies used a range of methods to study depression or anxiety with an analytic focus on language, discussions, and user interactions. Of these, 39% (7/18) of studies used ML, all of which entailed the use of unsupervised models with the exception of the Sharma and De Choudhury [66] study, which used supervised ML models to classify the degree of support exhibited in comments. Latent Dirichlet Allocation, an unsupervised ML model used to generate topics from large data sets of naturalistic texts, was most represented in 22% (4/18) of studies. A further 44% (8/18) of studies, although not using ML models, applied natural language processing (NLP) techniques to generate features or topics for analyzing mental health language and interactions. Among the 15 studies in this category that used ML and/or NLP, Reddit text was most often processed into features through assignment into categories from the Linguistic Inquiry and Word Count mental health lexicon (7/18, 39%) by using term frequency–inverse document frequency vectors (4/18, 22%) and by processing text into readability metrics (2/18, 11%). Of the 18 studies, 3 (17%) purely qualitative studies examined depression and anxiety in the context of Reddit discussion threads about rheumatoid arthritis [61], fathers’ experiences with postpartum depression [56], and the effectiveness of mobile mental health apps [40].

The analytic focus of studies that focused on language and interactions also ranged in scale. Approximately 67% (12/18) of studies analyzed subreddit-level language phenomena, including the Chakravorti et al [43] comparative study of trends in discussion topics in r/depression, r/anxiety, and r/suicidewatch from 2012 to 2018 and the Low et al [55] study, which used the COVID-19 pandemic as a point of reference for examining changes in discussion topics across 15 mental health subreddits from *prepandemic* versus *midpandemic* periods. Of the 18 studies, 3 (17%) demonstrated a user-level analytic focus on language. An illustrative example of this category is the Ireland et al [52] study of how 1409 users of the r/anxiety subreddit exhibited differences in language use when posting in r/anxiety versus other subreddits compared with a control group of users with no history of participation in r/anxiety. Finally, 11% (2/18) of studies analyzed language at multiple levels. These included the Park and Conway [59] study of written communication challenges encountered in Reddit’s mental health subreddits, which featured a subreddit-level linguistic analysis in addition to a longitudinal user-level analysis. Moving on from the analytic focus of included studies, Table 4 summarizes the implications for practice gathered from the discussion and conclusion sections of the included studies.

**Table 4:** Summarized practice implications (N=30).

Practice implication category and types		Included studies, n (%)	References
**Professional-focused implications (n=19)**			
	Interventions from health professionals	18 (95)	[42-44,48,52,63-65,68-70,72,74-77]
	Interventions from university counselors	1 (5)	[41]
**User-focused implications (n=9)**			
	Interventions to inform or direct users to information	4 (44)	[42,55,60,66]
	Interventions to prompt users to assess mental health risk	3 (33)	[54,58,67]
	Interventions to encourage comfort in discussing sensitive health topics	2 (22)	[62,73]
	Intervention to alter user text for improved readability	1 (11)	[59]
**Moderator-focused implications (n=6)**			
	Moderator-focused tool for sorting information about posts and users	6 (100)	[42,49,50,58,59,66]
**Patient education programs (n=3)**			
	Creation of formal patient education and support	3 (100)	[56,60,61]

### Practice Implications

Approximately 56% (30/54) of studies built on findings with one or more practice implications, most of which 63% (19/30) suggested incorporating insights from Reddit data into professional mental health practice. A demonstration of this idea was found in the Rao et al [64] study, which applied a neural network model to classify Reddit users as depressed. In discussing the implications of their study, Rao et al [64] highlighted the future possibility of “sensitive applications in combining clinical care with users’ online activities” [64]. Other studies were more specific in envisioning practice implications for clinicians as they described a future “clinical tool” [72] or a “diagnostic aid” [63] designed to bring Reddit data into professional contexts. However, others used the more generalized language of “mechanisms” [70], “automated processes” [74], “services” [42], and “resources” [43] in presenting implications motivated by enhancing professional mental health practice with the results of studying depression and anxiety using Reddit data.

Of the 30 studies, 9 (30%) suggested practice implications focused on Reddit users. Common among the user-focused implications were suggestions for interventions to direct users to information. The Sharma and De Choudhury [66] study, for example, used NLP techniques to measure concepts of accommodation and support in discussions on mental health subreddits. Sharma and De Choudhury [66] suggested that posts determined as receiving exemplary levels of accommodation and support be embedded into community guidelines for promoting “subconscious learning of the linguistic style of the community” [66] to assist users as they consider posting. Another strand of user-focused implications was proposed in the Park et al [60] study, in which Park et al [60] envisioned functionality designed to help users of Reddit’s mental health subreddits connect with other users discussing similar mental health issues and who share appropriate “contextual elements of experience” [60]. The Low et al [55] study similarly recommended an intervention to guide users identified as expressing mental distress to subreddit communities known for high levels of support or moderator activity.

Some studies pictured future applications of ML classification that are designed to enable Reddit users to self-assess their mental state. For example, in the discussion following the results of the Kim et al [54] multilabel classification study, Kim et al [54] envisioned a service which, with user consent, accesses a user’s post history to “provide the probabilities of each mental disorder” [54]. The Park et al [58] study of changes in language use among long-term users of the r/depression subreddit recommended a similar automated process that would continuously monitor the writing of individual users to detect “undesirable linguistic changes” and subsequently intervene to “raise self awareness of their changes of linguistic or emotional state” [58].

Of the 30 studies, 6 (20%) suggested practice implications for moderators of Reddit’s mental health subreddit communities, and ideas for improving moderation workflow through automated means were common among these. For example, Gkotsis et al [49] suggested the assignment of urgency markers for posts in need of timely moderator attention, and Sharma et al [66] recommended new tools to help the moderators of mental health subreddits “efficiently and quickly navigate the stream of incoming requests” [66]. Finally, 10% (3/30) of studies featuring practice implications suggested incorporating the study findings into formal patient education. To illustrate, the Park et al [61] qualitative study of depression and anxiety in the context of rheumatoid arthritis positioned study findings as potentially contributing to improved practical recommendations to guide health care within rheumatology. In summary, 56% (30/54) of the included studies mentioned practice implications that went beyond theoretical implications or recommendations for future research. Taken together, these practice implications give a lens through which the goals and imagined futures of this research area come into focus.

## Discussion

### Principal Findings

To the author’s knowledge, this scoping review is the first to map academic literature focused on the study of depression and anxiety using data from Reddit. The objective was to better understand the scope and nature of this research space by detailing its mental health conditions of focus, data collection approaches, analytic focus, and practice implications. The results showed comparatively more research attention directed to depression versus anxiety, an even split of original and premade data sets, a favored analytic focus on classifying the mental health states of Reddit users, and practical implications that frequently recommended new professionally driven monitoring and outreach for Reddit users. Researchers interested in advancing the study of depression and anxiety using Reddit data will benefit from further consideration of key insights and tensions contained within the main results, which are elaborated in the following 2 sections: (1) conceptual issues surrounding the interpretation of Reddit data with the medical model of mental health and (2) the importance of locating accountability and autonomy in practice implications suggesting new forms of engagement with Reddit users.

### Depression, Anxiety, and the Medical Model

Observations about the ways in which depression and anxiety were studied using Reddit data rest on 2 premises established at the outset of this review. First, the circulation of mental health information on Reddit is user driven and conceptually distinct from the domain of working medical professionals. Second, research practice works to transform Reddit from the naturalistic *communicative phenomenon* of social media into a *research object* through the application of research methodologies and the accumulation of academic knowledge [10]. This review shows the concepts of depression and anxiety on Reddit emerging as part of a research object that favors interpreting mental health problems through the medical model of practice and explanation [81]. The medical model was readily noticeable in generalizations about the nature of Reddit’s mental health subreddits through references to r/depression and r/anxiety as “clinical subreddits” [72] and the depictions of users accessing mental health subreddits as “diagnostic groups” [53], and “patients” [48,71]. Although these broad strokes of medical terminology are conjecture, they illustrate the medical model being put to work as an *interpretive frame* circumscribing grammar, conceptual boundaries, and claims to relevance in research [82]. The medical model has thus far proven to be influential as an interpretive frame; however, its influence appears to subsist in the absence of wider debate and negotiation. To bring understandings of depression and anxiety on Reddit into maturation, constructive thinking and discussion about the medical model as an interpretive frame will be needed.

A thought-provoking exception to implicit assumptions about the medical nature of Reddit’s mental health communities was noted in the Park et al [60] longitudinal study of thematic similarities and differences between r/depression, r/anxiety, and r/PTSD subreddits. Park et al [60] combined a topic modeling algorithm with qualitative analysis to analyze a total of 7410 posts and 132,599 comments made between January 2011 and December 2015. On the basis of comparative findings on the r/depression subreddit, such as its larger size and less active userbase, Park et al [60] hypothesized that “the word ‘depression’ perhaps has a larger set of connoted meanings, some clinical and others not; and thus, those who participate in this subreddit may be a more diffuse and transient group” [60]. With this observation, Park et al [60] suggested an alternative conceptualization of depression on Reddit that more resembles a communicative phenomenon and likely possesses far less clinical relevance. In recognizing this more expansive meaning of depression, Park et al [60] illustrated the medical model of mental health as just one way to approach the concepts of depression and anxiety in the context of social media data [4].

Although depression and anxiety can refer to categories of disorder diagnoses, they can also refer to symptoms of other mental health conditions, transient emotional expressions, or something else entirely. Borrowing an idea from the early 20th-century philosopher Wittgenstein [83], perhaps depression and anxiety on Reddit are best understood as *family resemblance concepts* with plural meanings that overlap, diverge, and shift over time on the scale of individual user expressions, discussion threads, and entire subreddit communities. Not only do these meanings elude clean division along the lines of professional versus lay knowledge, but it is also possible that ambiguity surrounding meanings of depression and anxiety can serve as a rhetorical resource in user-led discussions [84]. To this end, researchers seeking a broader understanding of what depression and anxiety mean for mental health information seekers on Reddit would benefit by incorporating openness to plural meanings of these terms into methodological choices. Although looking beyond the circumscriptions of the medical model may involve departing from the goal of accurately classifying user mental health states, it does not imply adversity to the analysis of big data sets using ML and DL. This difference was illustrated by some techniques charted in this review, such as Latent Dirichlet Allocation [44,46,55,57] and a relationship modeling network [47], which were used to investigate mental health language by processing naturalistic text in an unsupervised or bottom-up fashion, meaning that data from Reddit did not converge with any external lexicons or prelabeled data in processing. Unsupervised systems still reflect researcher choices and perspectives at other methodological decision points, such as the data collection approach, the tuning of algorithm parameters, and the interpretation of output; however, it is notable that unsupervised techniques avoid freighting the concepts of depression and anxiety with external information while still leveraging the analytic insight of ML. Qualitative research designs offer another methodological path to broaden the conceptualizations of depression and anxiety while also introducing limits to the scope and depth of analysis feasible for the *human instrument* in a single study. It stands that the qualitative designs included in this review were oriented to smaller, context-driven analyses, for example, understanding the meanings of depression in the context of other conditions such as rheumatoid arthritis [61].

In summary, provisional application of the medical model of mental health may be appropriate in certain approaches for studying depression and anxiety using Reddit data. One included study paraphrased the goal of preventing mental illness through predictive classification of social media data as wider progress toward “an unfulfilled promise of clinical science” [72]; this may be possible if ML approaches are carefully integrated with not only the terminology but also the human expertise and ground truth standards of the medical model. A likely explanation for the current dearth of substantive discussion about the conceptual foundations of the medical model in this research area is the interweaving of the medical model of mental health with the language and concepts of ML classification systems. The contingent of researchers potentially interested in wider conceptual engagement with the medical model extends fairly broadly into medical and social sciences; however, the specialized language of ML may act as a buffer against such engagement in this research niche [85]. The results of this review are hopefully encouraging, as they show that there is space for further examining the daring conceptual feat of importing established medical terminology into the novel algorithmic models of ML-driven studies of naturalistic Reddit data. More generally, the maturation of this research space will be served by putting the medical model into perspective as just one framework among many from which to comprehend the meanings of depression and anxiety with data from Reddit. The final section of the *Discussion* connects the conceptual issues arising from adherence to the medical model to ambitions for materializing tangible impacts on the mental health of Reddit users.

### Locating Accountability and Autonomy in Practical Implications

At the methodological center of the studies included in this review is a one-way flow of information, as researchers unobtrusively gather and analyze data sets of user expressions from Reddit related to depression and anxiety. User expressions eventually become the substrate of academic publications that leave no footprints in the web-based environments in which Reddit users originally participated. Given the ethical delicacy of this achievement, it is notable that the discussion sections of included studies frequently harbored the ambition to cross from a passive research practice into engagement with Reddit users through new professional digital outreach initiatives entailing monitoring and intervention [86]. In one sense, the horizon of eventual intervention lends purpose and a promise of future impact to studies of depression and anxiety using Reddit data. However, the digital outreach initiatives proposed in the included studies were understated in the positioning of Reddit—both the user base and the company—within as of yet unrealized sociotechnical configurations of academic researchers, ML systems designed to classify mental states, clinicians, and the larger digital wellbeing industry [87]. For researchers imagining new expressions of digital public mental health for Reddit users related to depression and anxiety, it will be useful to provide additional ideas for navigating the piecemeal, international, and commercially inclusive structure of the sociotechnical configurations involved. The idea of accountability in digital public health, summarized by Hoeyer et al [88] as “defining who needs to know—and do—what, and for and to whom” [88], would be a helpful anchor to ask the questions needed for advancing practice implications in this research area. For example, how would the scope of practice and incentive structure of professionals associated with a digital outreach initiative intersect with the accountability for the mental health needs and preferences of Reddit users? Relatedly, what would be the best way to incorporate algorithmic accountability of ML systems designed to initiate engagement with Reddit users or health professionals based on the classification of mental health states [89]? To work toward a more ethically sensitive foundation of accountability in decision-making about digital outreach, it will be necessary to consider these questions at the level of sociotechnical configurations. At a narrower level, researchers would also benefit from wading into the ethical dimensions of autonomy in the context of users seeking and sharing sensitive mental health information on Reddit.

The pseudonymous character of the user experience on the Reddit platform clearly grants a measure of autonomy for users to make relatively informed decisions about expressing sensitive mental health information. For researchers envisioning digital outreach for depression or anxiety based on monitoring the activity of Reddit users, valuing autonomy requires taking up the fundamental issue of whether Reddit users should be made aware that the signals of their participation in Reddit communities are being used as input features for the inference and assessment of mental health states. At stake is the very trust and disinhibition of Reddit users, which researchers identify as a merit of the platform as a mental health information environment [62]. Concerns related to monitoring are particularly consequential for Reddit users, as mental health states would be classified primarily through firsthand written expressions. As researchers tune the parameters of systems designed to monitor and classify written expressions of depression and anxiety that are actionable for some kind of outreach, they also inherit responsibility for deciding what constitutes proficient and mentally healthy written text. Digital outreach acting on assessments of naturalistic user text will inevitably be rooted in adherence to predetermined, standardized forms of communication that may not be applicable to users’ diverse communicative choices, abilities, and styles [90]. Furthermore, it would be wise not to underestimate the potential harms to individuals who become aware that a digital outreach system has labeled them as experiencing depression and anxiety. For example, a system for monitoring user text with a threshold for initiating digital outreach that is highly sensitive will generate false positives resulting in unknown harms to the users who become recipients of inappropriately deployed digital outreach [91]. It is also likely that harms would be sustained to some users whose mental state has been accurately classified as depressive or anxious but for whom digital outreach would be unwelcomed.

Studies of depression and anxiety using Reddit data have yet to amount to any tangible impacts for Reddit users; however, the intention to shift toward professional-facing and user-facing digital outreach was a common theme among the practice implications of the included studies. For researchers invested in realizing this variety of practice implications, there is a need to define accountability and locate it within the novel sociotechnical configurations that would be mobilized to deliver digital outreach. Stemming from the broad issue of accountability, practice implications in this research area would benefit from more substantial explorations of the meanings of autonomy in the context of depression and anxiety on the Reddit platform. Considering user autonomy from various angles will improve understandings of the nature of Reddit as an informational and social resource for depression and anxiety and, in turn, inform ethical deliberation regarding if, how, and by whom new digital outreach should be introduced.

### Strengths and Limitations

There were several strengths to this review. The broad inclusion criteria led to a relatively complete picture of the different ways in which depression and anxiety are currently being studied using Reddit data. Another strength was that the findings were synthesized and discussed with the intention of heightening awareness of the conceptual and ethical issues that can be challenging to apprehend in the context of individual studies but are, nonetheless, unfolding in relation to this research area. A key limitation of this review was that it excluded studies without an explicit methodological focus on depression and anxiety, and this decision was made for reasons of feasibility in addition to conceptual coherence. Therefore, studies with an exclusive focus on topics related to depression and anxiety, such as suicidality and eating disorders, were not considered in the findings. Another limitation was that this review captured many but not all studies related to the CLEF eRisk shared tasks on early depression detection. Researchers seeking to comprehensively capture all studies of depression using Reddit data in the future should include a *CLEF eRisk* concept in their systematic search strategy, as it appears that not all CLEF eRisk studies mention Reddit in the title or abstract. Finally, in accordance with the objective of this review, no formalized risk of bias assessment or quality appraisal steps were conducted. Such steps may be more appropriate in a systematic review focused on studies featuring a shared analytic focus or those using a specific method to study depression or anxiety using Reddit data.

### Conclusions

The objective of this review was to build an understanding of the scope and nature of research conducted using Reddit as a primary data source for studying depression and anxiety. A total of 54 studies were included for the review, and key features of methodological interest were communicated through tabular and descriptive means. The results demonstrated that studies of depression and anxiety using Reddit data are currently bound to a prevailing methodology that favors a technical, solution-based orientation. The discussion sheds perspective on this trajectory by highlighting the conceptual issues related to the medical model of mental health and the ethical issues pertaining to new forms of professional engagement with Reddit users for mental health prevention and treatment. This scoping review serves as a point of orientation as researchers navigate and build upon the landscape of research on depression and anxiety using Reddit data.

## Appendix

Multimedia Appendix 1 Ovid MEDLINE full search strategy.

## Abbreviations

API: application programming interface
CLEF: Conference and Labs of the Evaluation Forum
DL: deep learning
ML: machine learning
NLP: natural language processing
PRISMA: Preferred Reporting Items for Systematic Reviews and Meta-Analyses
PRISMA-ScR: Preferred Reporting Items for Systematic Reviews and Meta-Analyses Extension for Scoping Reviews
