# Supplementary material for: Studies of Depression and Anxiety Using Reddit as a Data Source: Scoping Review
Source: JMIR Ment Health. 2021 Nov 25;8(11):e29487. doi: 10.2196/29487 (PMC8663609; doi:10.2196/29487)
Supplement: Multimedia Appendix 1 [file mental_v8i11e29487_app1.docx]

| **#** | **Searches** | **Results** | **Annotations** |
| --- | --- | --- | --- |
| 1 | Reddit.tw,kf. | 200 | Reddit concept keyword |
| 2 | Anxi*.tw,kf. | 229235 | Anxiety concept keyword |
| 3 | Exp Anxiety/ | 86483 | Anxiety concept subject heading |
| 4 | Depressi*.tw,kf. | 418971 | Depression concept keyword |
| 5 | Exp Depression/ | 120833 | Depression concept subject heading |
| 6 | Mental Health.tw,kf | 167246 | Mental health concept keyword |
| 7 | Exp Mental Health/ | 39482 | Mental health concept subject heading |
| 8 | Mental Disorder*.tw,kf | 51337 | Mental disorder concept keyword |
| 9 | Exp Mental Disorders/ | 1250450 | Mental disorder concept subject heading |
| 10 | Mental illness*.tw,kf | 34404 | Mental illness concept keyword |
| 11 | 2 or 3 | 249148 | Anxiety concept combined searches |
| 12 | 4 or 5 | 443471 | Depression concept combined searches |
| 13 | 6 or 7 or 8 or 9 or 10 | 1381046 | Mental health concepts combined searches |
| 14 | 1 and 11 | 17 | Reddit + Anxiety concept |
| 15 | 1 and 12 | 21 | Reddit + Depression concept |
| 16 | 1 and 13 | 61 | Reddit + Mental health concepts |
| 17 | 14 or 15 or 16 | 67 | Combined |
